# A Three-Dimensional Method for Analysis of the Body Mode of Classical Guitars Using a Laser Displacement Sensor

**DOI:** 10.3390/s24165147

**Published:** 2024-08-09

**Authors:** Kuan-Cheng Su, Tsung-Yu Hsieh, Wei-Chih Lin, Fu-Li Hsiao, Tatyana Ryzhkova, Chii-Chang Chen

**Affiliations:** 1 Department of Optics and Photonics, National Central University, Jhung-Li, Taoyuan 320, Taiwan; kfcxx0218@gmail.com (K.-C.S.); ordinary35926@gmail.com (T.-Y.H.); twister0528@gmail.com (W.-C.L.); 2 Institute of Photonics, National Changhua University of Education, Changhua 500, Taiwan; fulihsiao@cc.ncue.edu.tw; 3 Independent Researcher, Sudweyher Str. 13, 28857 Syke, Germany; tatyanaguitarmail@gmail.com

**Keywords:** body mode, acoustic spectra, laser displacement sensor, timbre, classical guitars

## Abstract

In classical guitar acoustic spectra, the lowest frequency body mode’s amplitude often significantly surpasses that of the string overtones. However, the characteristics of the body mode have not been systematically utilized to quantitatively represent the timbre of classical guitars. In this study, we propose a quantitative method for describing the body mode, which can effectively differentiate the timbre of classical guitars. Our approach involves three key parameters presented in a three-dimensional space, as follows: the frequency and quality factors of the body mode, along with the amplitude ratio of the plucked string note to the body mode in the soundboard’s vibration spectrum. This representation allows for the visualization, quantitative comparison, and classification of the body mode note and damping properties across classical guitars. The differences in body mode among guitars can be analyzed quantitatively using Euclidean distance.

## 1. Introduction

The timbre of a musical sound, also known as tone quality or tone color, is assessed to differentiate between two notes played by different instruments or employing different performance methods [1,2]. A multidimensional scaling algorithm has been developed to quantitatively analyze the timbre of musical instruments. This algorithm estimates each timbre on Euclidean dimensions, facilitating a quantitative approach to timbre analysis [3]. A timbre space model was proposed for different musical instruments, employing five latent classes to create a three-dimensional spatial representation. Additionally, an alternative tristimulus method has been explored, utilizing the amplitude of the note and its overtones for analysis [4,5,6,7,8]. By employing a three-dimensional coordinate system where each axis corresponds to an aural attribute such as loudness, note, or attack time, the sounds of musical instruments can be effectively classified, visualized, and compared.

The lowest frequency body mode of the guitar [9], the so-called body mode, air resonance [10,11], Helmholtz resonance [12], or tuning note [11] of the instruments is the tone caused by the interaction between the soundboard and the air contained in the guitar body [13]. The body mode of the guitar could induce the wolf note [14]. The body mode of the guitar often lies in the span between E (at about 82 Hz) and A (at about 110 Hz) [11]. The properties of the body mode have been measured and investigated as Helmholtz resonators [11]. To the best of our knowledge, no literature on quantitative timbre analysis of musical instruments [1,4,15] takes into account the body mode of string instruments. However, this mode is characterized by its significant amplitude, and is solely dependent on the structure and materials of the guitars.

The amplitude of the body mode of the guitar body depends on the energy coupling efficiency between the string and the guitar body [16]. The quality factor of the body mode depends on the damping factor of the wood of the guitar body. From the point of view of the timbral perception, the amplitude ratio for the note of the plucked string and the body mode can be used to understand the importance of the body mode compared to the string note during their chorus. From the point of view of the physics, this ratio reflects the impedance of energy coupling via the bridge between the string and the guitar body, corresponding to the string-to-body impedance ratio [16]. In this work, we use the frequency and the quality factors of the body mode, as well as the amplitude ratio for the note of the plucked string and the body mode, to analyze the body mode of the guitars. By employing three quantitative parameters, the timbre of the guitar can be represented in a three-dimensional timbre space, enabling visualization, classification, and discussion of the perception of timbre.

In this study, a 1997 José Ramirez 3E guitar (soundboard: Cedar, side and back plate: Rosewood, weight: 1688 g, string length: 65 cm) and a hand-made classical guitar by our laboratory, featuring an asymmetrical bracing structure (soundboard: Sitka Spruce, side and back plate: Formosa Koa, weight: 1646 g, string length: 65 cm) with the dimension of Torres, were characterized. We chose a José Ramirez guitar because the luthier is well-known, and the guitars are often used as a reference for comparing the timbre of guitars. We selected our hand-made guitar because we can carefully control the structure, materials, and fabrication process. To investigate the body mode of the guitar without string, bursting balloons with precisely controlled inside pressure were used as the pulsed acoustic source inside the guitar body. For guitars with strings, the work (product of the displacement of the string and the pulling force) to excite the string is precisely controlled. To measure the vibration of the soundboard [17], in the literature, Laser Doppler Vibrometer (LDV) can provide resolution at the picometer level [18,19,20,21]. In this work, the laser displacement sensor with a sensitivity of 30 nm, which is available at a more affordable cost than LDV, is sufficient for measuring these parameters of the body mode.

## 2. Experimental Setup

**Figure 1:**
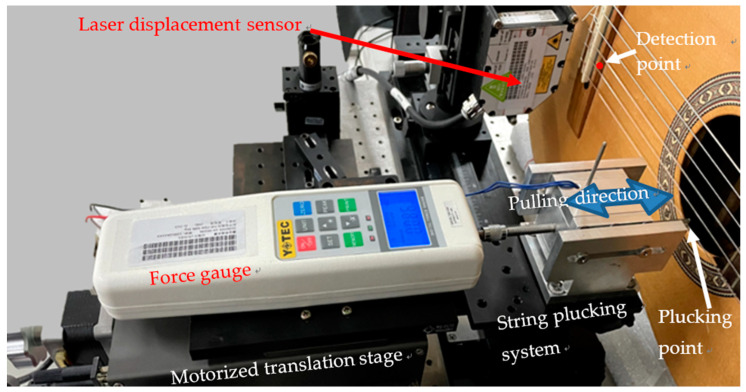
Laser displacement sensor and the string plucking system for excitation of the string vibration perpendicular to the soundboard. The hand-made guitar is mounted on the experimental setup. Forge gauge and motorized translation stage are installed to precisely control the pulling force and the displacement for plucking the strings.

In our study, the laser beam of the laser displacement sensor with the sensitivity of 30 nm is positioned on the bridge as indicated—“detection point” in Figure 1—to measure the displacement of the soundboard. The maximum displacement measurement range of the laser displacement sensor is 2 mm, which is sufficient for the displacement measurement of the guitar soundboard vibration. The power of the laser is less than 1 mW at the wavelength of 405 nm. The measurement is taken at the maximum sampling frequency of the laser displacement sensor to be 49 kHz, which is higher than the standard sampling rate for CD. The working distance between the laser displacement sensor and the soundboard is 10 mm, which is sufficient to prevent contact and damage to the guitar during the mounting operation. Figure 1 shows the laser displacement sensor and the hand-made guitar under test conditions.

The strings of Hannabach 600 MT (medium tension) and 600 HT (high tension) are installed on the guitars. The string plucking system consists of a computer-controlled motorized translation stage with the resolution of 1.25 μm, a force gauge, a pair of forceps, and a current-controlled electromagnet. The tip of the forceps is used to pull the string. The current-controlled electromagnet is connected to the forceps to relax the string without noise by turning off the current to excite the string into vibration. A force gauge of the sensitivity of 1 g is installed between the motorized translation stage and the forceps to measure the pulling force to the string. We use the computer to control the pulling force and the displacement of the string. The work to excite the guitar can be precisely obtained. The plucking position is located at 2/7 of the string length (18.6 cm) from the bridge. This position is above the sound hole where the guitarists often pluck the strings. The plucking excitation at this position suppresses the 7th, 14th, and 21st, etc., overtones of the string vibration. The fundamental note and the 2nd to 6th ordered overtones could be excited. Figure 1 also shows the string plucking system for the excitation of the string vibration perpendicular to the soundboard.

The classical guitars consist of two main resonant structures, as follows: the guitar body, made of wood and the nylon strings. To study the coupling of two resonant structures, measuring the resonant spectra of each structure individually can help identify the origins of the resonant peaks in the spectra obtained from the coupled structures. The influence on the resonance after the coupling of the two structures can then be observed. To characterize the body mode of the guitar without strings, a balloon with precisely controlled pressure of 2.2 psi is placed inside of the guitar body through the sound hole. By piercing the balloon, the pulsed acoustic sound is generated. The pulsed acoustic sound inside the guitar body can more efficiently excite the vibration of the guitar body than the loudspeaker outside the guitar body [11]. All the measurements of the soundboard displacement in this study are recorded for 20 sec. The Fourier transform is applied to the temporal displacement data to obtain the vibration spectrum. The corresponding spectral resolution is 0.1 Hz.

## 3. Results and Discussion

**Figure 2:**
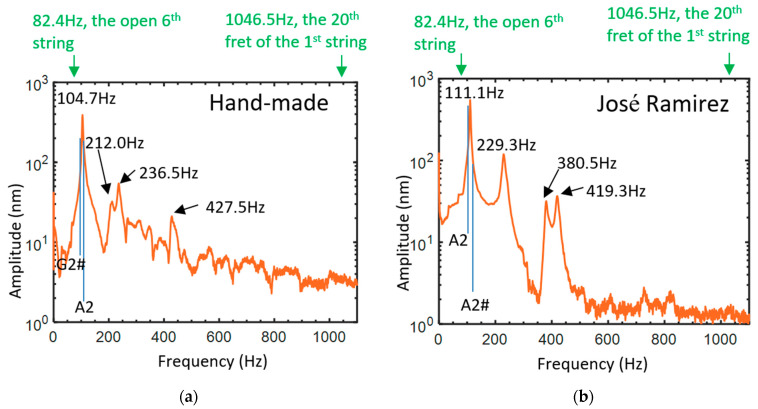
Spectra of the body modes for (**a**) the hand-made guitar and (**b**) the José Ramirez guitar without installation of string.

The spectra of the body mode of the guitar measured with pulsed acoustic sound are shown in Figure 2a,b for the hand-made guitar and the José Ramirez guitar, respectively, without installation of the string. We can find the strongest amplitude of the body modes located at 104.7 Hz and at 111.1 Hz for the hand-made guitar and the José Ramirez guitar, respectively. The frequency range of the main notes for the guitars is indicated in Figure 2, from 82.4 Hz (open sixth string) to 1046.5 Hz (the twentieth fret of the first string). The body mode of the hand-made guitar at 104.7 Hz with the full-width at half-maximum (FWHM) of 5.4 Hz is close to the frequency of the G2# note (the fourth fret of the sixth string), at 103.8 Hz for the A440 tuning standard. The body mode at 111.1 Hz with the FWHM of 5.1 Hz for the José Ramirez guitar almost coincides with the frequency of the A2 note at 110 Hz, which is also the note of the open fifth string for the guitar. The quality factors of the body modes are 19.4 and 21.8 for the hand-made and the José Ramirez guitar, respectively, showing the slightly higher damping property for the hand-made guitar. The note of the body mode can last longer for the José Ramirez guitar. The standard error of the amplitude of the body mode is 7% for 10 measurements, showing the good stability of the measurement using the excitation of the bursting balloon.

**Figure 3:**
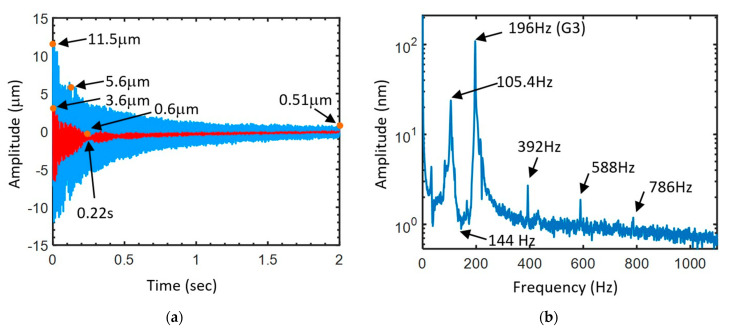
(**a**) The blue curve presents the amplitude variation of the soundboard by plucking the third string perpendicularly to the soundboard of the hand-made guitar. We applied a low-pass filter at the frequency of 144 Hz on the blue curve to obtain the red curve, representing the significant amplitude variation of the soundboard for the body mode of the guitar. (**b**) The spectrum of the amplitude variation of the soundboard of the hand-made guitar.

The blue curve of Figure 3a shows the exponential decay of the vibration of the soundboard by plucking the third string perpendicularly to the soundboard of the hand-made guitar. The displacement of the plucking point on the string is 2 mm, which usually happens during the performance of guitarists. The work applied on the string is 2.97 mJ. The initial amplitude of the vibration of the soundboard is 11.5 μm. For a duration of 2 s after the string excitation, the amplitude decays to 0.51 μm. The attenuation of elastic potential energy is calculated by $10\times {log}_{10}(A_{1}^{2}/A_{0}^{2})$, where $A_{0}^{2}$ and $A_{1}^{2}$ are the amplitude squared at time t_0_ and t_1_, respectively, to be −27 dB. The duration of 2 s of a note corresponding a full measure at the tempo of 120 beats per minute (bpm) usually happens in some guitar music. In our experiment, the sound after 2 s can still be well heard. A sixteenth note with the duration of 0.125 s at the tempo of 120 bmp can often be performed by guitar musicians. The amplitude at 0.125 s is 5.6 μm. The attenuation of the elastic potential energy is −6.3 dB.

In Figure 3a, the red curve presents the variation of the amplitude of the body mode after a low-pass filtering of the frequency above 144 Hz to observe the pure vibration of the body mode. During the initial vibration, the amplitude of the red line is around 1/3 of the blue line, showing the fact that the sound volume of the body mode is important. However, the decay of the red line is also significant compared to that of the blue line. The first minimum of the note is found in 0.22 s, indicating that the sound of the body mode might be almost extinguished after or before the duration of two sixteenth notes (0.25 s) for the hand-made guitar. The corresponding attenuation of the elastic potential energy is −13.4 dB. If the note duration of the body mode (quality factor) with significant amplitude can last longer, less changes of the timbre happen during the note duration. Figure 3b presents the corresponding spectrum of Figure 3a. We can observe that the body mode is located below 144 Hz. The amplitude of the body mode is much more important than the overtones at 392, 588, and 786 Hz. Therefore, the characteristics of the body mode should be considered as the quantitative parameters of the guitar timbre.

**Figure 4:**
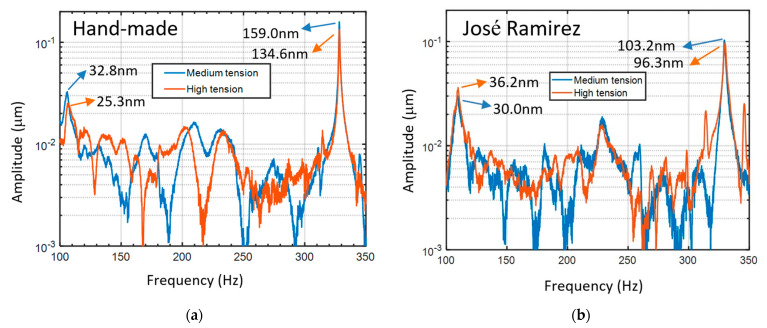
Acoustic spectra of (**a**) the hand-made guitar and (**b**) the José Ramirez guitar by plucking the open first string perpendicularly to the soundboard. The blue and red lines present the spectra by installing the medium tension strings (Hannabach 600 MT) and the high tension strings (Hannabach 600 HT), respectively.

To investigate the acoustic mechanism or compare the timbre of musical instruments, a specific note should be chosen. For example, in Ref. [4], the C4 note (261.6 Hz) was selected to compare the timbre of organs and violas. To study the body mode (wolf note), the G-string of violins was excited [14]. For guitar music, melodies are often played on the first string. Therefore, to characterize the peak of the body mode, the first string of the guitars is plucked perpendicularly to the soundboard. Figure 4a,b illustrate the vibration spectra of the hand-made guitar and the José Ramirez guitar with medium (Hannabach 600 MT) and high tension strings (Hannabach 600 HT). The work for plucking is fixed to be 7.92 mJ for all measurements. The displacement of the string and the pulling force on the string are listed in Table 1. The amplitudes of the body mode are 32.8 nm and 25.3 nm for the medium and the high tension strings, respectively, for the hand-made guitar. The amplitudes of the body mode are 30.0 nm and 36.2 nm for the medium and the high tension strings, respectively, for the José Ramirez guitar. We calculate the ratio of the amplitude of the note of the first string (A_p_) to that of the body mode (A_r_). A_p_/A_r_ is 4.85 and 5.32 for the medium and the high tension strings, respectively, for the hand-made guitar. For the José Ramirez guitar, A_p_/A_r_ is 3.44 and 2.66 for the medium and the high tension strings, respectively. The ratio of A_p_/A_r_ of the hand-made guitar is obviously larger than that of the José Ramirez guitar, revealing that the bass component (body mode) in the timbre of the first string is more important for the José Ramirez guitar. Although a specific note is typically chosen to investigate the acoustic mechanism or compare the timbre of musical instruments, in our study, we took measurements of the acoustic spectra of guitars by exciting all strings. The spectra obtained by exciting the fifth string were used to analyze the strong coupling between the string and the guitar body [22]. Only negligible differences were found between the body modes of the other spectra. Therefore, in this study, we present only the spectra obtained by exciting the first string, where the melody is often played.

**Figure 5:**
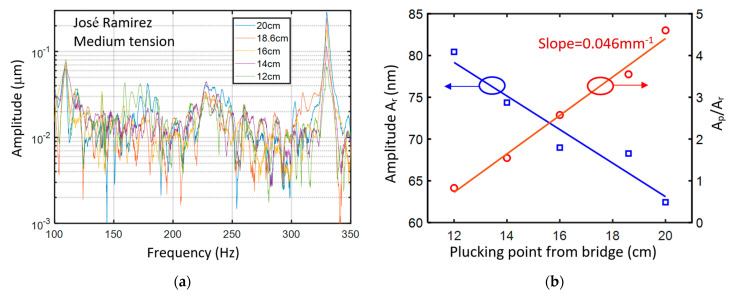
(**a**) Vibration spectra of the José Ramirez guitar by plucking the first string perpendicularly to the soundboard at different positions from the bridge. (**b**) Blue squares and red circles present the amplitude of the body mode (A_r_) and A_p_/A_r_, where A_p_ is the amplitude of the note of the first string. Solid lines are the linear curve fitting results.

To study the influence of the plucking position on the string to the amplitude of the body mode, A_r_, and the ratio A_p_/A_r_, the medium tension first string of the José Ramirez guitar is plucked perpendicularly to the soundboard at the positions that are 12 to 20 cm from the bridge. Figure 5a shows the vibration spectra. We can observe that A_r_ decreases and A_p_ increases as the plucking position varies from 12 to 20 cm with respect to the bridge. Figure 5b presents A_r_ and A_p_/A_r_ for the different plucking positions. A_r_ decreases approximately linearly as the distance from the bridge increases. A_p_/A_r_ also increases linearly as the distance from the bridge increases. We adopted a linear curve fitting for A_p_/A_r_, as shown by the red solid line. The slope of the red solid line is 0.046 mm^−1^. Our accuracy for the plucking position is 1 mm. The ratio of A_p_/A_r_ in Figure 4 is around five and three for the hand-made and José Ramirez guitars, respectively. An uncertainty of 0.046, corresponding to an approximate 1% error for measuring A_p_/A_r_, could be acceptable for quantitatively distinguishing the timbre.

To illustrate the body mode, the coordinates of the body mode for the two guitars are drawn in the three-dimensional space. The three axes of the three-dimensional space are the frequency and the quality factor of the peaks of the body mode, as well as the ratio A_p_/A_r_, as the first string was plucked at the position of 18.6 cm from the bridge. The lower or higher body mode frequency represents colder or more brilliant bass, respectively. The higher quality factor represents the lower damping factor of the wood and the longer sustentation of the bass. The lower ratio A_p_/A_r_ represents the higher intensity or the stronger bass sound. These characteristics that are originated from the properties of the pure guitar body can be used to define the timbre of the guitars.

In the methods for analyzing the timbre of guitars [4,5,6], the spectral centroid was obtained by calculating the centroid of the overtones weighted by their corresponding amplitudes [6]. The tristimulus method divides the frequency spectrum of the acoustic signal into three bands. The first band represents the amplitude of the fundamental note. The second band represents the sum of the amplitudes of the second, third, and fourth overtone components. The third band represents the sum of the amplitudes of the higher overtone components [4]. For plucked string instruments such as guitars, it is well known that the spectral envelop of the overtones can be precisely calculated based on the excitation position on the strings [7]. Both methods utilize the amplitudes of the overtones from the acoustic spectra, which can indeed be mathematically predicted based on the excitation (plucked) position for string instruments such as guitars [7,8]. This observation suggests that under identical plucking conditions, the timbres of different guitars could exhibit slight variations. Both methods are not suitable to distinguish the timbre of the guitars.

**Figure 6:**
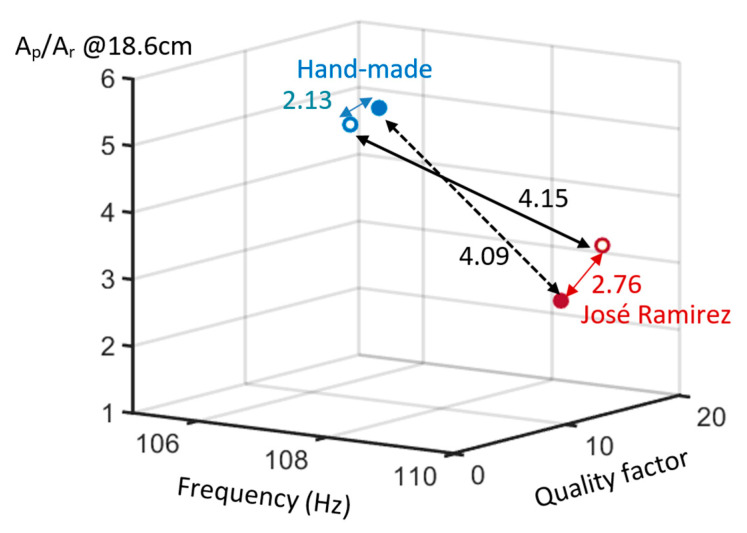
The coordinates in the three-dimensional space for the hand-made and José Ramirez guitars. The axes are the frequency and the quality factors of the peaks of the body mode as well as the ratio A_p_/A_r_, as the first string was plucked at the position of 18.6 cm from the bridge. Open circles present for the medium tension string. Solid circles present for the high tension string.

In Figure 6, we can observe that the frequency of the body mode is lower for the hand-made guitar. The standard deviation of the frequency of the body mode is 0.14. The quality factors of the peaks of the body mode, which depend on the ratio of the modal masses of the string and the structure [9], are 13.6 and 11.0 for the hand-made guitar with the medium and high tension strings, respectively. For the José Ramirez guitar, the quality factors are 14.8 and 12.9 for the medium and high tension strings, respectively. The standard deviation of the quality factor measurement is 0.7. The fact that the quality factor of the body mode for the guitar with strings, shown in Figure 5a, is lower than that without strings, shown in Figure 2, is noted. The quality factor of a system consisting of the two vibration cavities with the quality factors of Q1 and Q2 can be expressed by 1/Qtotal = 1/Q1 + 1/Q2 [23]. In our case, Qtotal, Q1, and Q2 are the quality factors of the body mode for the guitar with strings, the quality factor of the body mode for the guitar without strings, and the quality factor of the strings, respectively. From the expression, Qtotal is dominated by the minimum of Q1 and Q2. It can be proven mathematically that Qtotal is lower than Q1 and Q2. Therefore, the quality factor of the body mode for the guitar with strings is lower than that without strings. With this expression, we can deduce that the quality factors of the string are 45.5 and 25.5 for the medium and the high tension strings, respectively, from the experimental data of the hand-made guitar. From the experimental data of the José Ramirez guitar, the quality factor of the string can be found to be 46.3 and 31 for the medium and the high tension strings, respectively. The results are approximately consistent.

The Euclidean distances between the two guitars are shown in the three-dimensional space to be 4.15 and 4.09 for the medium and the high tension strings, respectively. For the hand-made and the José Ramirez guitars, the Euclidean distances between the coordinates for different strings are 2.13 and 2.76, respectively. In fact, with the same strings, the timbre difference between the two guitars under test conditions can be obviously distinguished. This difference can also be illustrated using the Euclidean distance in the three-dimensional space. This analysis can used to quantitatively compare the similarity between the timbres of the classical guitars.

## 4. Conclusions

In this work, we propose to measure the ratio of A_p_/A_r_, as well as the frequency and the quality factor of the peaks of the body mode in the vibration spectrum, to quantitatively analyze the body mode of the classical guitars. The timbre of the guitar can be represented in a three-dimensional timbre space, enabling visualization, classification, and discussion of the perception of timbre. The results obtained from a 1997 José Ramirez 3E guitar and a classical guitar hand-made by our laboratory were compared. The frequency of the body mode is lower for the hand-made guitar, providing a cold or firm timbre. The quality factor of the body mode is lower for the hand-made guitar, displaying its slightly higher damping property. In the linear acoustic system [24], A_p_ is proportional to A_r_. Both A_p_ and A_r_ are proportional to the excitation force. Using A_r_ as the parameter of the body mode may introduce additional errors due to inaccuracies in the excitation force. The advantage of using the ratio of A_p_/A_r_ as the parameter of the body mode instead of A_r_ is to normalize and compare with the amplitude of the string note. The importance of the bass (body mode) in the timbre of the first string can be described. The second advantage is the fact that the ratio does not depend on the work applied on the string. The results obtained from different research groups can be compared. Using the Euclidean distance in the three-dimensional space, the similarity between the timbres of the classical guitars can be quantitatively analyzed.

## Figures and Tables

**Table 1:** The displacement and the pulling force of the first string.

Guitar	String Tension	Displacement	Pulling Force	Work
José Ramirez	Medium	4 mm	1.98 N	7.92 mJ
	High	3.78 mm	2.09 N	7.92 mJ
Hand-made	Medium	4.17 mm	1.90 N	7.92 mJ
	High	3.92 mm	2.02 N	7.92 mJ

## Data Availability

The datasets used and/or analyzed during the current study are available from the corresponding author on reasonable request.
